# Porphyrin-Modified Polyethersulfone Ultrafiltration Membranes for Enhanced Bacterial Inactivation and Filtration Performance

**DOI:** 10.3390/membranes15080239

**Published:** 2025-08-06

**Authors:** Funeka Matebese, Nonkululeko Malomane, Meladi L. Motloutsi, Richard M. Moutloali, Muthumuni Managa

**Affiliations:** Institute for Nanotechnology and Water Sustainability, College of Science, Engineering and Technology, University of South Africa, Florida, Johannesburg 1709, South Africa; 69504857@mylife.unisa.ac.za (N.M.); 66466393@mylife.unisa.ac.za (M.L.M.); moutlrm@unisa.ac.za (R.M.M.)

**Keywords:** bacterial inactivation, porphyrins, municipal wastewater, ultrafiltration membranes

## Abstract

Municipal wastewaters pose a severe risk to the environment and human health when discharged untreated. This is due to their high content of pathogens, such as viruses and bacteria, which can cause diseases like cholera. Herein, the research and development of porphyrin-modified polyethersulfone (PES) ultrafiltration (UF) membranes was conducted to improve bacterial inactivation in complex municipal wastewater and enhance the fouling resistance and filtration performance. The synthesis and fabrication of porphyrin nanofillers and the resultant membrane characteristics were studied. The incorporation of porphyrin-based nanofillers improved the membrane’s hydrophilicity, morphology, and flux (247 Lm^−2^ h^−1^), with the membrane contact angle (CA) decreasing from 90° to ranging between 58° and 50°. The membrane performance was monitored for its flux, antifouling properties, reusability potential, municipal wastewater, and humic acid. The modified membranes demonstrated an effective application in wastewater treatment, achieving notable antibacterial activity, particularly under light exposure. The In-BP@SW/PES membrane demonstrated effective antimicrobial photodynamic effects against both Gram-positive *S. aureus* and Gram-negative *E. coli*. It achieved at least a 3-log reduction in bacterial viability, meeting Food and Drug Administration (FDA) standards for efficient antimicrobial materials. Among the variants tested, membranes modified with In-PB@SW nanofillers exhibited superior antifouling properties with flux recovery ratios (*FRRs*) of 78.9% for the humic acid (HA) solution and 85% for the municipal wastewater (MWW), suggesting a strong potential for long-term filtration use. These results highlight the promise of porphyrin-functionalized membranes as multifunctional tools in advanced water treatment technologies.

## 1. Introduction

Water scarcity and quality have become pressing global concerns, affecting millions of people worldwide. Contributing factors include, but are not limited to, climate change and ongoing population growth [1,2,3]. In particular, microbial waterborne diseases like cholera, typhoid, and diarrhea are some of the leading causes of death globally [4,5]. As such, the increasing demand for clean water, coupled with the growing threat of waterborne diseases, necessitates the development of effective and sustainable water treatment technologies. Among the various methods employed for water treatment, membrane filtration has become a promising approach due to its ability to remove various contaminants, including viruses, bacteria, and particulate matter [6,7].

Polyethersulfone (PES) ultrafiltration (UF) membranes are commonly employed in wastewater treatment because of their thermal stability and outstanding mechanical and chemical resistance [8,9]. PES membranes remove microorganisms via advanced filtration processes while improving water quality [10,11]. However, PES membranes suffer microbial accumulation, ultimately leading to biofilm formation and biofouling, which can significantly compromise their efficiency and lifespan [12,13,14]. The development of antimicrobial membranes that can effectively inactivate or remove microorganisms is crucial for ensuring the production of safe drinking water [11,15,16]. The overall aim is to improve the performance of the membrane without altering desired properties such as stability, mechanical strength, and a prolonged lifespan.

Porphyrins, a class of macrocyclic compounds, have garnered considerable interest owing to their distinctive photophysical as well antimicrobial characteristics [17,18]. Upon light activation, porphyrins generate reactive oxygen species (ROS) that damage cellular components, finally resulting in microbial inactivation [19]. Porphyrins, when metalated with diamagnetic heavy metals such as indium, show improved stability and singlet oxygen quantum yields, ultimately leading to improved antibacterial activity [20,21]. Notably, porphyrins have improved the antimicrobial properties of polyacrylonitrile membranes, reaching 85.9 and 99.9% against *Staphylococcus aureus* (*S. aureus*) and *Escherichia coli* (*E. coli*), respectively [22]. This demonstrated the potential of these materials for anti-biofouling. Moreover, single-walled carbon nanotubes (SWCNTs) alone were shown to enhance the antimicrobial characteristics of polymer membranes, such as those made from polyamide [23]. In addition to this, previous studies indicate that SWCNTs alone have an improved antibacterial efficiency when conjugated to porphyrins as compared to when used as a standalone against various bacteria, including *E. coli* and *S. aureus* [24,25]. However, no study exists on the use of porphyrins in conjugation with SWCNTs for improving properties of PES membranes.

Building on these findings, this study is the first, to our knowledge, to report the incorporation of novel metal-free and indium(III) derivatives of 5,10,15-tris-(4-bromopheyl)-20-(4-carboxyphenyl)porphyrin alone and conjugated to SWCNTs into PES membranes to create antimicrobial UF membranes against *S. aureus* and *E. coli*. This study aims to provide insights into the possibility of porphyrin-based PES membranes for the treatment of water, offering a promising solution for producing safe drinking water.

## 2. Materials and Methods

Pyrrole (98%) was purified by distillation prior to use. Tetrahydrofuran (99.9%), propionic acid (ACS reagent, ≥99.5%), isopropanol (99.5%), glacial acetic acid (100%), 4-bromobenzaldehyde (ReagentPlus, 98%), 4-formylbenzoic acid (97%), indium(III) chloride, chloroform (100%), sodium acetate, dimethyl formamide (ACS reagent 99.8%), methanol (MeOH, ACS reagent, ≥99.8%), 1-methyl-2-pyrrolidinone (NMP), polyethersulfone powder (PES), and ethanol (ACS reagent, ≥96%) were sourced from Sigma Aldrich (Darmstadt, Germany).

### 2.1. Synthesis of 5,10,15-Tris(5-bromophenyl)porphyrin (BP) as Well as Its Corresponding Indium Chloride Derivative ClIn(III), 5,10,15,Tris-(4-bromophenyl)-20-(4-carboxyphenyl)porphyrin (In-BP)

BP was synthesized following a modified Alder–Longo method [26,27], were 4-formylbenzoic acid (1.5 g, 10 mmol), 4-bromobenzoic acid (6.6 g, 36 mmol), as well as pyrrole (0.66 mL, 9.5 mmol) were refluxed in propionic acid (150 mL) for 3 h at 150 °C. The reaction was monitored to completion using UV-Vis Spectrophotometry. The porphyrin was air-dried and subjected to purification by silica gel column (hexane/tetrahydrofuran) and then dried in air.

For the metalation of free base porphyrin, BP (242 mg, 0.27 mmol) was first dissolved in glacial acetic acid (10 mL) and then added into a refluxing mixture that contained acetic acid (40 mL) and sodium acetate (112 mg, 1.35 mmol). Indium(III) chloride (442 mg, 2.00 mmol) was subsequently added, then the reaction continued for 4 h. Completion of the metalation was determined via UV-Vis Spectrophotometry. The mixture was left to cool at ambient temperature and subsequently recrystallized using sodium hydroxide (NaOH).

BP Yield (43.23%) FTIR: -N-H (3633 cm^−1^), -O-H (3306 cm^−1^), -C-H (2852–2920 cm^−1^), -C=O (1703 cm^−1^), -C=N (1592 cm^−1^), -C-N (1401 cm^−1^), -C-O (1158 cm^−1^), and Br-C (1067 cm^−1^). MALDI-TOF MS *m/z* calc: 895.45 found [M-H] 895.41. UV-Vis (DMF) λmax nm (log ε): 416, 513, 546, 587, and 643.

In-BP Yield (89.98%) FTIR: -N-H (3670 cm^−1^), -O-H (3430 cm^−1^), -C-H (2852–2920 cm^−1^) -C=O (1687 cm^−1^), -C=N (1602 cm^−1^), C-N (1401 cm^−1^), (-C-O) (1208 cm^−1^), and Br-C (1010 cm^−1^). MALDI-TOF MS *m/z* calc: 1043.70 found [M-Cl] 1002.034. UV-Vis (DMF) λmax nm (log ε):426, 560, and 600.

### 2.2. Conjugation 5,10,15-Tris(5-bromophenyl)porphyrin (BP) as Well as Its Corresponding Indium Chloride Derivative ClIn(III), 5,10,15,Tris-(4-bromophenyl)-20-(4-carboxyphenyl)porphyrin (In-BP) with Single-Walled Carbon Nanotubes (SWCNTs)

The fabrication of porphyrin single-walled carbon nanotubes was carried out with modifications following literature procedures [25,28]. A 0.2 mM porphyrin solution in DMF (10 mL, spectral grade) was mixed with 0.5 mg of SWCNTs, and the mixture was sonicated (using a SonicClean, LaboTec, Midrand, South Africa) for 4 h at room temperature (RT). To get rid of the insoluble carbon nanotubes, the solution was spun in a centrifuge for approximately 1 h after that.

### 2.3. Fabrication of Membranes

The flat-sheet membranes were fabricated via the NIPs method. PES polymer (18 wt.%) and NMP solvent (82 wt.%) were stirred in a beaker at RT until the polymer was totally dissolved. The prepared solution was desiccated overnight to remove gas bubbles. A 200 μm airgap casting knife was employed to apply the casting solution evenly across the glass plate. The class plate was immersed in water to allow the coagulation process to take place. The membranes were kept in DI water at 4 °C up to the time of use. Then, a bare PES membrane was prepared. The modified PES membranes were prepared by adding 18 wt.% PES polymer, 0.25 wt.% of nanofiller, and NMP solvent (81.75 wt.%), as shown in Table 1, to prepare a dope solution. Similar casting steps were followed for membrane casting.

### 2.4. Characterization Methods

Fourier Transform Infrared (FT-IR) Spectroscopy (Perkin Elmer, Waltham, MA, USA, FT-IR 100) was employed to identify functional groups (FGs) in the synthesized materials and membranes. The ATR method was used for porphyrins as well as conjugates (4000–530 cm^−1^). The Ultraviolet-Visible (UV-Vis) absorption spectra were measured with a PerkinElmer UV/Vis/NIR spectrometer Lambda 1050 (Perkin Elmer, Waltham, MA, USA). For nanoparticles and conjugates, solid-state UV-Vis spectra were collected over a range of 250–800 nm, while porphyrins dissolved in DMF were analyzed using liquid-state UV-Vis spectra in the 300–800 nm range. Fluorescence emission spectra were acquired by a Varian Eclipse spectrofluorometer. For fluorescence emission measurements, a time-correlated single photon counting (TCSPC) system (FluoTime 300, PicoquantR GmbH, Berlin, Germany) was employed, equipped with a diode laser (LDH–P–C-420, 420 nm, 20 MHz repetition rate, 44 ps pulse width), following a previously established procedure. Mass spectrometry was conducted by Brucker AutoFLEX III smartbeam TOF/TOF Mass spectrometer, Bruker Daltonics, Billerica, MA, USA, which uses a positive ion mode alpha-cyano-4- hydroxycinnamic acid in a MALDI matrix. Contact angle, which is used to study surface wettability of the membranes, was obtained at RT on the surface energy evaluation system Data-Physics Optical Contact Angles (OCAs) 15 EC (G10, KRUSS, Hamburg, Germany), which includes video recording capabilities. The membrane surface and cross-section morphology were examined using scanning electron microscopy (SEM) (TESCAN VEGA 3, TESCAN, Brno, Czech Republic) operated at an acceleration voltage of 20 kV. Atomic force microscopy (AFM, Nanoscale IV, Veeco, New York, NY, USA) was utilized to measure the surface roughness of the membranes.

### 2.5. Membrane Performance Assessment

The membrane water uptake and porosity capabilities were evaluated by soaking the membrane in DI for 24 h followed by drying the membranes under air for 24 h. The membrane’s weight was measured before soaking, while they were wet. The membranes’ water uptake and porosity (*ε*) were estimated by means of Equations (1) and (2), respectively.

(1) $$Water\;uptake\;\left(\%\right)=\frac{M_{wet}-M_{dry}}{M_{dry}}\times 100\;$$

(2) $$\varepsilon \;\left(\%\right)=\frac{M_{wet}-M_{dry}}{\rho_{H_{2}O}\times A\times \delta }\times 100\;$$

where $M_{wet}$ signifies weight of a wet/damp membrane (kg), $M_{dry}$ is the mass of a dry membrane (kg), *ρ* signifies water density (kg m^−3^), *δ* signifies membranes cross-sectional length (m), and *A* signifies membranes area (m^2^) [29].

The Guerout–Elford–Ferry (*r_m_*) Equation (3) was utilized to calculate the average pore size of the membranes [30]:

(3) $$r_{m}=\sqrt{\frac{(2.9-1.75\varepsilon )8\eta \delta Q}{\varepsilon \times A\times ∆P}}\;$$

where *η* signifies water viscosity (8.9 × 10^−4^ Pa·s), *r_m_* signifies average pore radius (nm), *ε* signifies membrane porosity, ∆P signifies operating pressure (MPa), and *Q* signifies volume of permeated water per unit time (m^3^/s).

The membrane performance was assessed using a dead-end cell. The fabricated membranes were compacted at 250 kPa until a constant flow was achieved. Pure water flux (PWF, *J_flux_*) was evaluated at 180 kPa for 5 min. Equation (4) was used for calculating PWF (*J_flux_*) [31,32],

(4) $$J_{flux}=\frac{Q}{t\times A}\;$$

where *J_flux_* presents the membranes permeation flux in (L·m^−2^ h^−1^), *t* signifies permeation time (h), *A* signifies area of the membrane fitted in the dead-end cell (m^2^), and *Q* stands for the volume of the permeate (*L*).

The rejection (*R*) performance of the membranes was determined by HA. The HA concentration in the feed and permeate was analyzed using a UV-Vis spectrometer, and Equation (5) was utilized to calculate the rejection by membranes [33].

(5) $$R\left(\%\right)=\left(1-\frac{C_{p}}{C_{f}}\right)\times 100\;$$

where *R* signifies the rejection, *C_f_* signifies feed solution concentration, and *C_p_* signifies the permeate concentration.

The fouling resistance properties of the fabricated membranes were investigated by filtration of pure water (PW) to obtain PWF (*J_w,a_*) and filtration of a 1000 ppm humic acid (HA) solution and municipal wastewater to obtain their flux (*J_p_*). The membranes were backwashed with PW at 200 kPa to remove attached foulants via shear force. Pure water was filtered through the cleaned membranes to obtain another PWF (*J_w,b_*). The membrane fouling was evaluated in detail using the following parameters, namely, flux recovery ratio (*FRR*), total fouling (*R_t_*), reversible fouling (*R_r_*), and irreversible fouling (*R_ir_*). Equations (6)–(9) were utilized to derive the listed fouling parameters, respectively [34,35].

(6) $$FRR=\left(\frac{J_{w,b}}{J_{w,a}}\right)\times 100\;$$

(7) $$R_{t}=\left(1-\frac{J_{p}}{J_{w,a}}\right)\times 100\;$$

(8) $$R_{r}=\left(\frac{J_{w,b}-J_{p}}{J_{w,a}}\right)\times 100\;$$

(9) $$R_{ir}=\left(\frac{J_{w,a}-J_{w,b}}{J_{w,a}}\right)\times 100\approx R_{t}-R_{r\;}\;$$

### 2.6. Municipal Wastewater Sampling

The raw municipal wastewater was collected from the Mzimkhulu Water Treatment Plant in uMzimkhulu, KwaZulu-Natal Province, South Africa, and carried to the laboratory in airtight bottles. Turbidity, total dissolved solids (TDS), pH, and electrical conductivity (EC) parameters were used to analyze the water before and after treatment. The quality of the effluent was compared with the South African National Standard (SANS 241). The multiparameter potable meter equipped with pH/EC/TDS/turbidity sensor probes was sourced from HANNA Instruments, Johannesburg, South Africa.

### 2.7. Antimicrobial Photodynamic Inactivation (aPDI)

*E. coli* and *S. aureus* were grown on agar plates based on the manufacturer’s specifications to isolate individual colonies. A single colony of each strain was transferred into 5 mL of fresh sterile Luria broth and then incubated at 37 °C with shaking at ~200 rmp for 18–24 h. Samples were periodically collected to monitor optical density until mid-logarithmic phase was reached between 0.6 and 0.8. The bacteria cells were centrifugated at 3000 rpm for 15 min and washed with Phosphate-Buffered Saline (PBS) three times. This process was followed by resuspending the cells in PBS at pH 7.4 and counting viable colonies for freshly prepared *E. coli* and *S. aureus* bacteria cultures were determined by serially diluting the cultures (10^−4^–10^−9^). Triplicate 100 µL aliquots from each dilution were plated on agar and incubated for at 37 °C for 24 h. The viable bacteria colonies were counted on a Scan 500^®^ series Automatic Colony Counter (Thorlabs, Newton, NJ, USA). Optimized bacterial suspensions with concentrations of 2.78–3.01 × 10^8^ CFU mL^−1^ for *S. aureus* and 1.62–2.01 × 10^8^ CFU mL^−1^ for *E. coli* were prepared for further experiments.

For photoirradiation, M420L4 Thorlabs LED was utilized for 30 min, while another set of 24-well plates were kept in the dark for the same duration to study vitro dark cytotoxicity. Following an altered technique [36,37], 1 cm membranes were cut and placed on the 24-well plate containing the bacteria and supplemented with 100 μL of freshly prepared broth. The plates were then incubated at 37 °C for 18 h. To evaluate treatment effects on bacterial cells, 10 μL of resazurin assay reagent was added to each well, followed by an additional 3 h incubation. Fluorescence readings (λex = 560 nm, λem = 590 nm) were taken in triplicates using Synergy 2 multimode microplate reader (BioTek^®^, Agilent, Santa Clara, CA, USA. To determine the bacterial cell survival percentage, Equation (10) was used with results presented as mean ± standard deviation.

(10) $$\%\;Cell\;Viability=\frac{Absorbance\;of\;sample\;at\;540\;nm}{Absorbance\;of\;control\;at\;540\;nm}\times 100\;$$

## 3. Results and Discussions

### 3.1. Nanofiller Characteristics

#### 3.1.1. FTIR and Mass Spectrometry

**Scheme 1:**
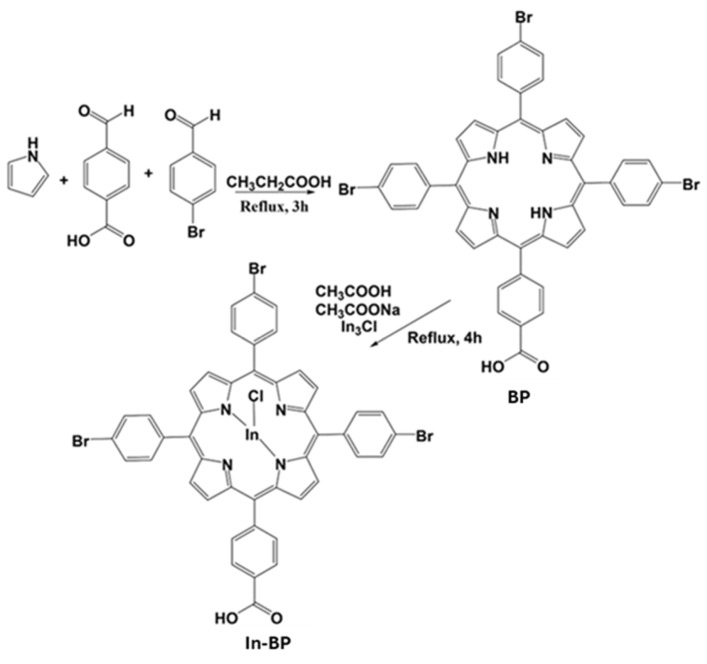
Synthesis of BP and In-BP.

**Scheme 2:**
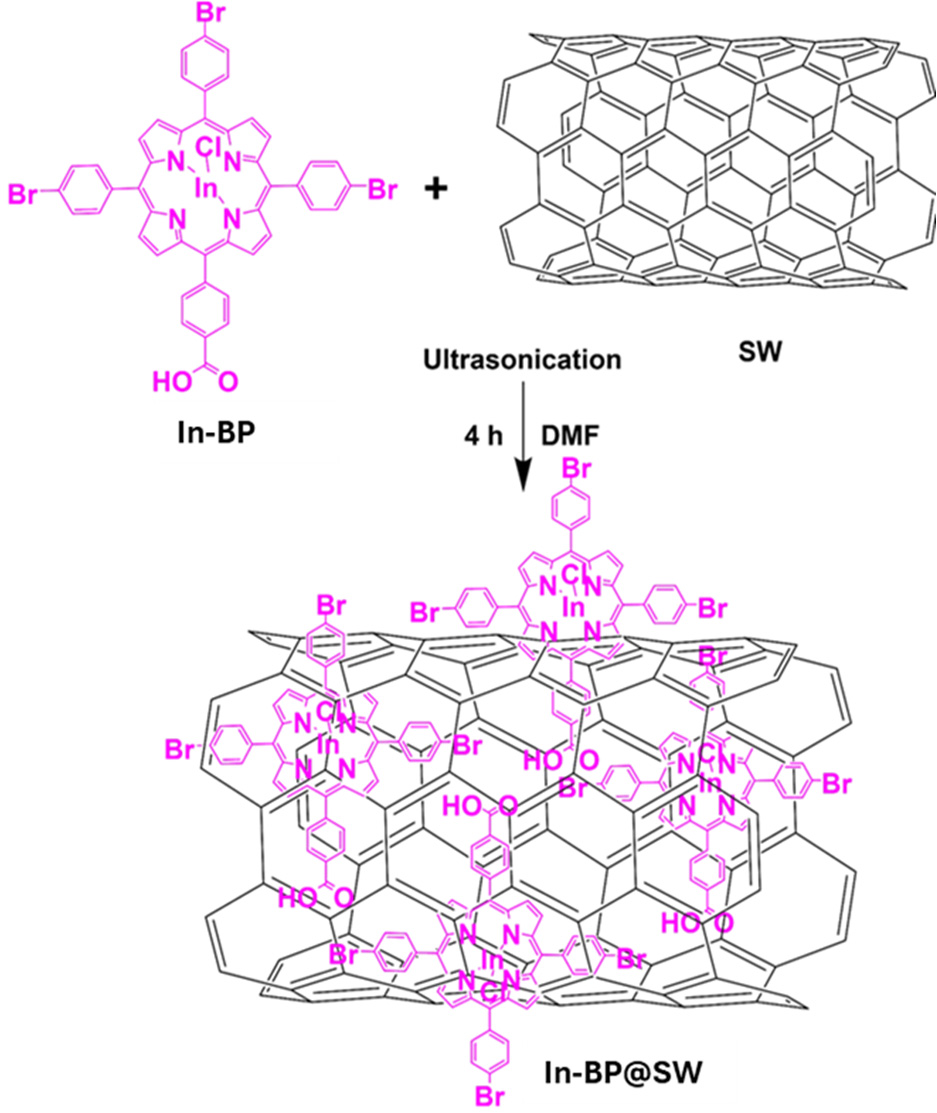
The fabrication of porphyrin single-walled carbon nanotubes.

Scheme 1 and Scheme 2 shows the synthesis of the BP, In-BP, and In-PB@SW as well as their structural confirmation was determined by FTIR and MALDI-TOF MS (Figures S1–S3). As seen on Figures S2 and S3 in the Supporting Information, MALDI-TOF MS exhibited molecular ion peaks at m/z = 895.41 and 1002.034 [M-Cl], corresponding to BP and In-BP, respectively. FTIR (Figure S1) confirmed the functional groups present in BP and In-BP, with a C=O group at 1703 and 1687 cm^−1^, respectively, while the C-O stretching vibrations appear around 1208 cm^−1^. Additionally, peaks corresponding to the -C-N of the pyrrole ring are seen at 1408 cm^−1^, indicating the presence of this functional group as also previously reported regarding the functional groups of porphyrins [38,39].

#### 3.1.2. UV-Vis Spectrophotometry

**Figure 1:**
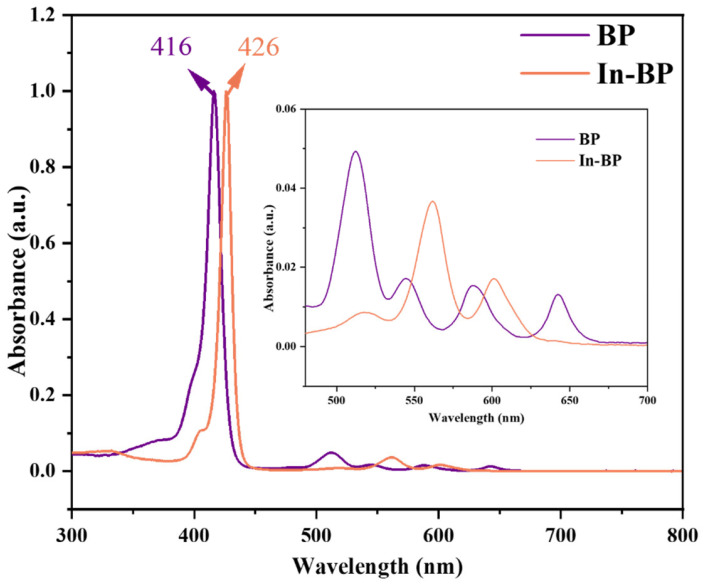
UV-Vis absorption spectra of BP and In-BP in DMF.

The UV-Vis was employed to determine the light absorbance of the synthesized materials. Figure 1 depicts the absorption peaks for BP and In-BP measured between 300 and 800 nm in DMF. The results showed that the porphyrin (BP) and its indium metalated complex (In-BP) exhibited distinct absorption peaks within the visible light spectrum. BP displayed a prominent Soret band at 416 nm, while Q bands were found at 514, 551, 591, and 647 nm, respectively. After metalation, the Soret band shifted 10 units to the red region, while the Q bands merged into two Q band peaks at 564 and 604 nm. These findings align with previous reports on indium(III) porphyrins and are attributed to the heavy metal effect, which alters the porphyrin structure and enhances intersystem crossing [25].

#### 3.1.3. Fluorescence Emission

**Figure 2:**
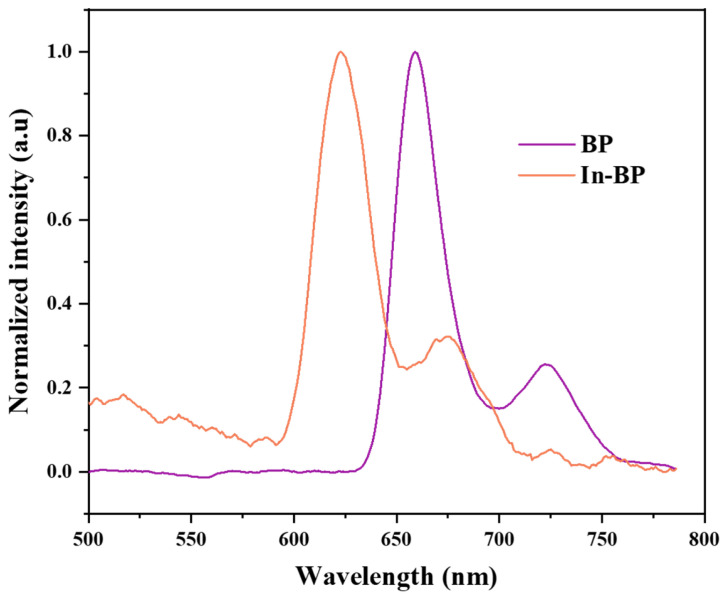
Fluorescence emission spectra of BP and In-BP in DMF (excited at 490 nm).

Fluorescence emission spectra were measured in DMF with an excitation of 43 nm. Figure 2 displays the S1→S0 fluorescence of BP as well as In-BP, revealing a clear distinction in their emission profiles. All the complexes show two typical fluorescence peaks of porphyrins. The metal-free porphyrin BP displayed robust and prominent peaks near 660 nm for Q(0,0), accompanied by secondary peaks which are less intense at 725 and 722 nm for Q(0,1) for BP and In-BP, respectively. In contrast, there was an observed blue-shift in the emission profile of In-BP, with peaks appearing at 622 nm (strong intense) and 676 nm (less intense). These differences in the emission wavelengths may be due to remarkable altercations in the porphyrin macrocycle’s photophysical characteristics and electronic structure, probably influenced by the indium metal center affecting the ligand’s excited-state dynamics [25,40].

### 3.2. Membrane Characteristics

#### 3.2.1. Fourier Transform Infrared Spectroscopy

**Figure 3:**
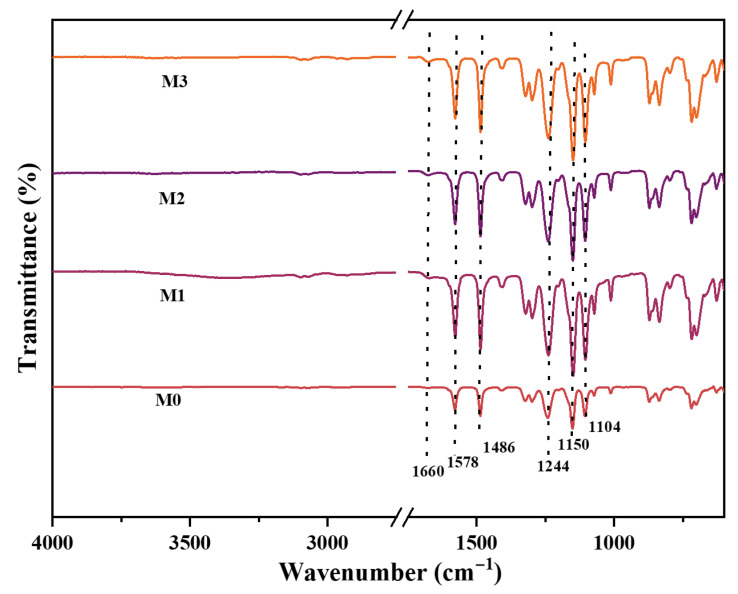
FTIR of membranes M0–M3.

The functional groups of the fabricated membranes were analyzed using FTIR, with the resulting spectra presented in Figure 3. The bare PES membrane exhibited all the expected characteristic peaks, namely, the aromatic ether at approximately 1244 cm^−1^, 1,4-disubstituted aromatic benzene rings at 1486 and 1578 cm^−1^, and the sulfonate appearing in the range of 1150 and 1104 cm^−1^. All the modified membranes exhibited similar peaks as the bare PES membranes, with an additional peak appearing at approximately 1660 cm^−1^. The new peak was assigned to the carboxylic acid (C=O) functionality of the porphyrins [41].

#### 3.2.2. Water Contact Angle, Porosity, and Pore Size Measurements

**Figure 4:**
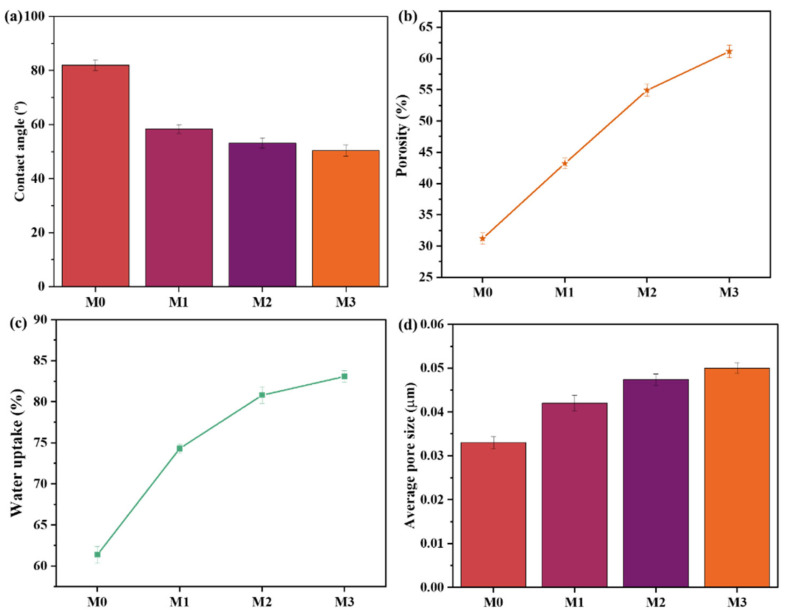
(**a**) Contact angle, (**b**) porosity measurements, (**c**) water uptake, and (**d**) average pore size of M0–M4 membranes.

The membrane’s affinity towards water was investigated via the contact angle (CA), water uptake, porosity, and average pore size methods, as depicted in Figure 4. For the CA (Figure 4a), the bare PES membrane (M0) depicted the highest water CA of 82°. The M1 membrane exhibited an improved surface hydrophilicity with a CA of 58°. The improved surface properties are due to the presence of the hydrophilic FGs (-COOH) possessed by the In-BP. The In-BP and In-BP@WS-modified membranes (M2 and M3) exhibited more hydrophilic surfaces with CAs of 53° and 50°, respectively. The SWCNTs possess a hollow tubular structure with a larger surface area containing abundant water-loving FGs, such as -COOH and -NH_2_, providing channels for water to pass through and increasing the membrane’s affinity to water [42]. The water uptake and porosity of the fabricated membranes were investigated to further evaluate the hydrophilic properties of the membranes. Both the porosity (Figure 4b) and water uptake (Figure 4c) followed an increasing trend after the membrane modification. The M0 membrane exhibited the lowest porosity and water uptake of 31.18 and 61.3%, respectively. The most hydrophilic surface was presented by the M3 membrane with a porosity of 61.0% and a water uptake of 83%. The Guerout–Elford–Ferry equation was utilized to determine the mean pore size (Figure 4d) of the membranes. The average membrane sizes ranged between 0.033 and 0.050 µm. The modified membranes exhibited improved hydrophilic surfaces with a decreasing CA. The improved membrane’s affinity toward water is attributed to the hydrophilic properties of different forms of the porphyrin added [43,44].

#### 3.2.3. Scanning Electron Microscopy and Atomic Force Microscope (AFM)

**Figure 5:**
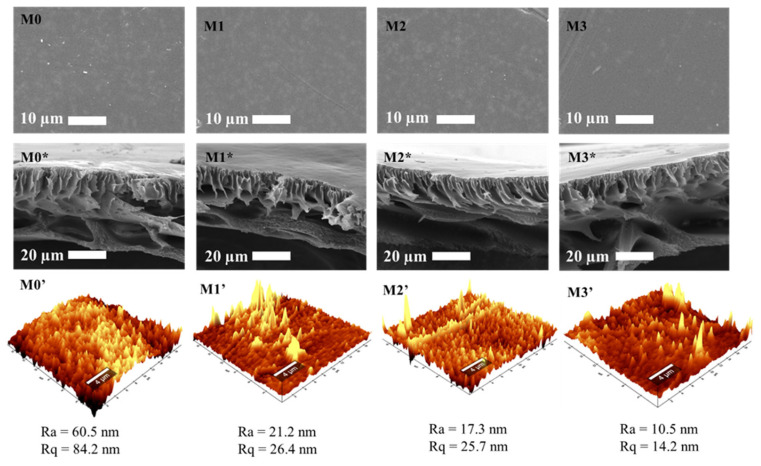
SEM (top surface: M0–M3 and cross-section: M0*–M3*) and AFM (M0’–M3’) images showing the surface morphology of the fabricated membranes.

The membrane’s surface morphology (top surface and cross-section) was evaluated using SEM, and the findings are depicted in Figure 5. The SEM analysis was important for examining their structure, pore size, and pore distribution. Additionally, the membrane structure plays a critical role in influencing the fouling behavior, hydrophilicity, and filtration performance [45,46,47]. The membranes presented a smoother and more porous surface with visible macropores clearly presented after the membrane dissection. The porous membrane surfaces are due to the solvent–nonsolvent exchange process, which results in pores depending on the exchange rate [48]. The M0 membrane presented fewer pores relative to the nanofiller-modified membranes. The modified membranes exhibited a larger porous surface and large pore distribution, which is ascribed to the incorporation of the hydrophilic properties contributed by porphyrins. Similar findings were reported in the literature [49]. The cross-sectional images exhibited two morphologies. The top layer shows macrovoid structures, which confirms the presence of the inner pores within the membrane. The sublayer is made of a sponge-like material with small pores clearly visible. The thickness of the sublayer decreased with an increase in the membrane hydrophilicity [41]. The 3-D AFM images and roughness parameters (Ra—average roughness and Rq—root mean square of the z-data) attained from the scan area of 10 μm × 10 μm for the fabricated membranes are also shown in Figure 5. The images have dark and bright points, which represent the valleys and peaks of the membrane surfaces. The membrane surface degree determines the behavior of the fouling resistance during filtration; the rougher surfaces tend to trap pollutants, thereby increasing the fouling tendency of the membranes. The M0 membrane has exhibited a relatively higher surface roughness of 60.5 nm, which can negatively impact its selectivity, fouling resistance, and flux. The modified membranes, M1-M3, exhibited a decreasing roughness (21.2–10.5 nm), which suggests that the incorporated nanofiller had a positive influence on the membrane surface. These outcomes are good for the overall performance of the membranes, especially the fouling resistance, because it suggests longer operational periods and extended lifespans of the membranes [50,51].

### 3.3. Membrane Application 

#### 3.3.1. Flux

**Figure 6:**
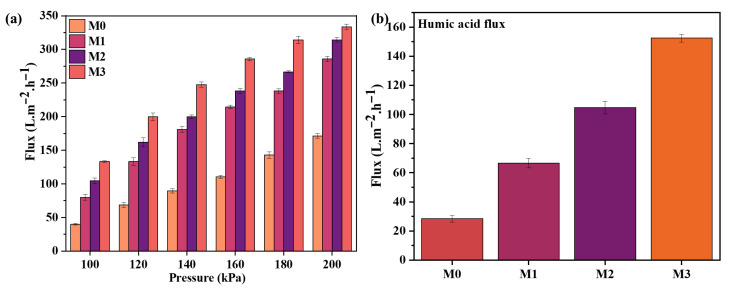
(**a**) Pure water flux at transmembrane pressure ranging from 100 to 200 kPa; (**b**) humic acid solution flux at 200 kPa.

The membrane permeation was assessed using pure water and a 1000 ppm HA solution (Figure 6). The pure water was permitted to pass through the surface of the membranes under various pressures varying from 100 to 200 kPa (Figure 6a). The fabricated membranes showed a trend that increases with an increase in the transmembrane pressure applied. The M0 membrane exhibited the lowest flux, ranging between 40.0 and 142.9 Lּּּּּּּm^−2^ h^−1^. A noticeable increase in the PWF was observed following the modification of the PES membrane with different porphyrins. The flux exhibited a rising pattern in the sequence M1 < M2 < M3. Specifically, the PWF improved from a range of 80.0 and 285.7 L·m^−2^·h^−1^ in M1 to 104.8 and 314.3 L·m^−2^·h^−1^ in M2 and further to 133.3 and 333.3 L·m^−2^·h^−1^ in M3. The HA permeation was assessed at 200 kPa (Figure 6b). All membranes exhibited a lower permeation for HA relative to their PWF. The M0 membrane showed a PWF of 89.5 L·m^−2^·h^−1^ and an HA flux of 28.6 L·m^−2^·h^−1^. For the modified membranes, the M1 membrane exhibited a PWF of 181.0 L·m^−2^·h^−1^ and an HAF of 66.7 L·m^−2^·h^−1^, M2 showed a PWF of 200.0 L·m^−2^·h^−1^ and an HAF of 104.8 L·m^−2^·h^−1^, while the M3 membrane recorded the highest values with a PWF of 247.6 L·m^−2^·h^−1^ and an HAF of 152.4 L·m^−2^·h^−1^ under the same operating conditions. The decline in the HAF is attributed to the accumulation of protein foulants on the surface of the membranes, causing pore narrowing and blockage. This is in line with what was previously reported in the literature [52,53].

#### 3.3.2. Fouling Resistance Reusability Potential of Membranes

**Figure 7:**
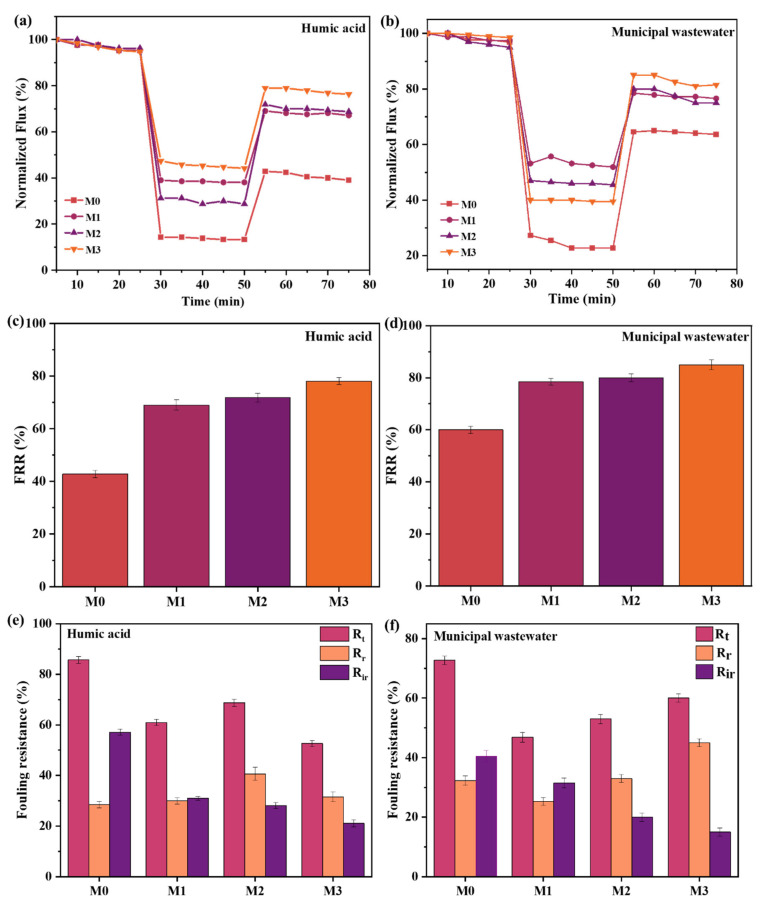
Normalized (**a**) humic acid and (**b**) municipal wastewater flux and (**c**,**d**) flux recovery ratio and (**e**,**f**) fouling resistance of humic acid and municipal wastewater, respectively.

**Figure 8:**
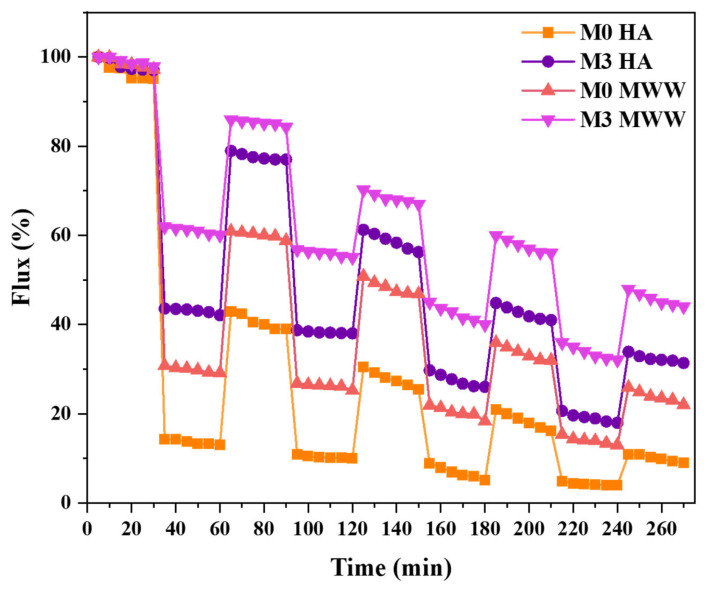
Reusability potential of M0 and M3 membranes for HA and MWW.

The fouling resistance abilities of the membranes (M0–M3) were evaluated using a 1000 ppm HA solution and real municipal wastewater (MWW). The test procedure involved an initial filtration of pure water through the membranes, followed by the filtration of either HA or MWW at 200 kPa for 25 min to induce fouling. Subsequently, the fouled membranes were backwashed with pure water at a higher pressure (200 kPa) to dislodge strongly adhered contaminants on the membrane surface and inside the pores. After backwashing, the pure water was filtered again to complete one antifouling cycle. Figure 7 depicts the obtained results. The M0 membrane demonstrated a poor antifouling performance compared to the modified membranes after filtration with both HA and MWW (Figure 7a,b). The M0 membrane showed a low *FRR* of 42.8 and 60% following the HA and MWW fouling, respectively (Figure 7c,d). The flux of M1 is larger than that of M2 between 30 and 50 min because membrane M2 possesses enhanced antifouling properties relative to M1. This minimizes the stubborn attachment to foulants of the surface of the membranes, resulting in more reversible fouling than irreversible fouling. Although M1 exhibited a lower hydrophilicity compared to M2, it possesses a loss matric, resulting in a higher flux. However, due to its relatively higher surface roughness, M1 is more prone, hence there is a decline in the flux after 50 min. This behavior suggests that the M2 membrane has better long-term antifouling characteristics than the M1 membrane. In contrast, the modified membranes exhibited significantly improved *FRRs*, with the In-BP@SW-modified membrane (M3) showing the highest recovery: 78.9% for HA and 85% for MWW. During filtration of municipal wastewater, membrane M3 exhibited relatively lower flux than that of M1 and M2 between 30 and 50 min because M3 has a higher density of active sites and a tighter pore structure compared to M1 and M2 membranes. These characteristics promote greater foulant accumulation on the membrane surface during the initial stages of filtration, leading to a temporary decline in the flux. However, once equilibrium is reached, the membrane’s performance stabilizes, aided by the excellent antifouling properties of the incorporated nanofillers. This trend is evident in the reusability studies shown in Figure 8.

Furthermore, the antifouling performance was assessed through reversible fouling (*R_r_*), total fouling (*R_t_*), and irreversible fouling (*R_ir_*), as depicted in Figure 7e,f. These are the key concepts used to better understand the membrane performance. *R_t_* represents the total flux decline (*R_r_* and *R_ir_*) caused by the foulant accumulation on and within the membrane surface during the filtration process. *R_r_* happens when foulants can be easily removed from the membrane surface by means of backwashing in this case. In contrast, *R_ir_* refers to foulants that cannot be washed off the membrane surface or pores, often requiring the use of harsh chemicals for cleaning, and this rapidly deteriorates the membrane’s structural integrity. The degree of these fouling types depends on the membrane’s characteristics [54,55]. The M0 membrane exhibited the highest *R_t_* (85.7%) after the HA filtration, with 28.5% *R_r_* and 57.2% *R_ir_*. A similar trend was observed after the MWW filtration, where the M0 membrane showed the highest *R_t_* (72.7%), comprising 32.7% *R_r_* and 40% *R_ir_*. The higher *R_t_* of the M0 membrane is due to its rougher surface (Ra—60.5 nm). During the HA and MWW filtration, foulants were trapped on the surface or within the membrane pores. The backwashing with water was therefore ineffective, as the *R_ir_* was higher. The modification of the PES membrane resulted in smoother surfaces, which aided the filtration process; foulants on the membrane surface were more easily removed during backwashing.

Additionally, hydrophilic surfaces help delay the deposition of foulants onto the membrane surface. Thus, the best-performing membrane (M3) demonstrated significantly reduced fouling, with an *R_t_* of 52.6 and 60% after the HA and MWW filtration, respectively. The *R_r_* accounted for 31.6% (HA) and 45% (MWW), while *R_ir_* was limited to 21.1% (HA) and 15% (MWW), confirming its superior antifouling capabilities [56,57]. In Figure 7f, M3 exhibited the highest *R_r_* because of its surface properties, which are improved hydrophilicity and a smoother surface morphology. The good antifouling properties possessed by M3 lead mostly to reversible fouling, unlike the more strongly adsorbed or embedded foulants on M1 and M2. The degree of fouling is also influenced by the nature of foulants present in the feed solution. In Figure 7f, the antifouling performance of the membranes was assessed using raw municipal wastewater. The M3 membrane showed a higher total fouling, primarily due to its tighter pore structure, which led to the greater accumulation of foulants and suspended solids on the membrane surface. However, these deposits were largely removable through physical cleaning, such as backwashing.

The reusability potential of the fabricated membranes was studied due to its importance in predicting operational reliability, cost-effectiveness, and environmental sustainability. Four fouling–cleaning cycles were performed using domestic wastewater and humic acid, with the results presented in Figure 8. The membranes used for these tests are the reference membrane (M0) and the best-performing membrane (M3) for both HA and MWW. The M0 membrane exhibited low reusability potential, as the *FRR* remarkably decreased to 10.1% and 23.9% for HA and MWW, respectively, after the fouling–cleaning cycles. This clearly indicates a reduced flux and separation efficiency, which is attributed to the hydrophobic nature of the material, as reported in the literature [44,58]. As a result, these unmodified membranes require frequent cleaning (e.g., backwashing or chemical cleaning), which often compromises the integrity of the membrane structure. This leads to poor separation and severe fouling, necessitating the replacement of the membrane. Thus, the frequent cleaning and replacement of the membrane consumes more resources, increasing the operational and maintenance costs of the treatment system. In contrast, the modified membranes have shown totally different outcomes, as expected [46,59,60,61]. Specifically, the incorporated nanofillers enhanced the membrane characteristics. These membranes require less cleaning and replacements compared to the unmodified membranes, as they are less exposed to harsh chemical cleaning for longer. This makes modified membranes more desirable for real-world treatment systems. The M3 membranes exhibited the *FRR* values of 32.4% and 45.7% for HA and MWW, respectively, after the last cleaning process.

#### 3.3.3. Municipal Wastewater Characteristics and Quality

The quality of the raw municipal wastewater and the effluent after the filtration was evaluated based on the pH, total dissolved solids (TDS), turbidity, and electrical conductivity (EC) (Table S1). The results were compared to the effluent discharge standards SANS: 241. The pH of the raw MWW was 7.04, while the pH of the membrane-treated water samples was 7.54, 7.41, 7.73, and 7.67 for M0, M1, M2, and M3, respectively. Both the feed and the permeate fell within the acceptable pH range (5–9.5) according to the SANS: 241. The slight change in the pH was noticed and attributed to the removal of the weakly acidic and basic substances or ion exchange interactions at the membrane surface. The raw MWW exhibited a turbidity of 43.64 NTU. This is because the municipal wastewater contains large amounts of suspended solids, compromising the turbidity of the water. After filtration, the turbidity of the permeates was reduced to 0.87 NTU (M0), 0.14 NTU (M1), 0.04 NTU (M2), and 0.00 NTU (M3), all below the permissible limit (≤5 NTU). The turbidity of the permeate exhibited a decreasing trend from M0 to M3, suggesting an improvement in membrane selectivity upon modification. The raw MWW exhibited a high EC of 767 µS/cm, which was higher than the acceptable limit (≤170 µS/cm). High EC in water is an indication of a high ionic content in the feed water. After the filtration with the pristine PES membrane M0, the EC reduced to 549 µS/cm. A further decrease in the EC was observed after the filtration of MWW with modified PES membranes (M1–M3), ranging between 392 and 251 µS/cm, which was still higher than the permissible limit. The TDS exhibited similar trends to those of the EC. The raw water exhibited the highest TDS of 549 ppm, followed by M0 with a TDS of 341 ppm, then M1 with a TDS of 282 ppm, and then M2 and M3 with a TDS of 231 and 159 ppm, respectively. The quality of both the feed and the membrane-filtered permeates was within the acceptable TDS limits (≤1200 ppm). The decrease in the overall turbidity, EC, and TDS highlights the membranes’ ability to remove suspended, colloidal, and biological particles primarily by size exclusion. The improved removal efficiency of the modified membranes is likely due to a combination of the size exclusion and the electrostatic interactions. These results suggest that the incorporation of the metalated conjugated porphyrins can enhance the membrane selectivity and antifouling properties, making them promising candidates for advanced wastewater treatment applications [62,63,64,65,66].

#### 3.3.4. Bacterial Inactivation

**Figure 9:**
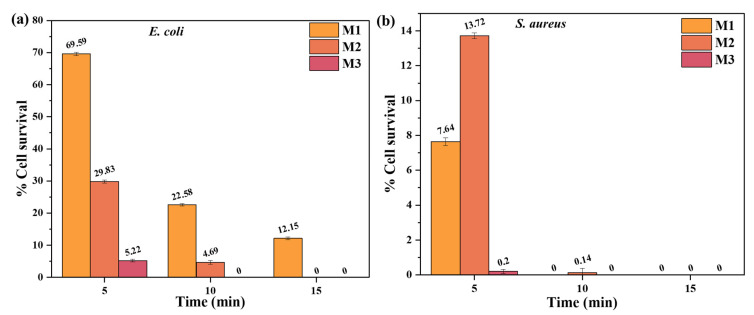
(**a**) % cell survival of *E. coli* bacteria after inactivation and (**b**) % cell survival of *S. aureus* bacteria after.

The antibacterial characteristics of the pristine and porphyrin-based membranes were investigated against *S. aureus* gram (+) and *E. coli* gram (−) strains. The results obtained in this study are illustrated in Figure 9a,b and Table 2 and Table 3. The %cell survival of both *E. coli* and *S. aureus* decreases with time, indicating a decrease/reduction in the initial percent of bacteria. According to the FDA, which recommends that at least a 3-log reduction in bacteria must be obtained for a photosensitizer to be regarded as good in microbial inactivation, all photosensitizers used in this study qualify as good photosensitizers [67], as above 3-log reductions were obtained. However, an ideal photosensitizer is one that can inactivate all bacteria within a short space of time. A recent study reported the antimicrobial photodynamic capabilities of SW in conjugation with indium(III) 5,10,15,20-tetrakis(4-pyridyl)porphyrin, which demonstrated impressive activity, reaching a 9.76- and 8.38-log reduction in *S. aureus* and *E. coli*, respectively [25]. Close results were achieved in this study as well, reaching 9.79- and 8.03-log reductions in *S. aureus* and *E. coli*, respectively. SW inactivates bacteria by causing mechanical damage to bacterial cell membranes and cell walls and oxidative stress, while porphyrins inactivate by singlet oxygen under light. As a gram (−) bacterium, *E. coli* is generally known to be difficult to inactivate with neutral photosensitizers. However, within 10 min, membrane M3 (In-BP@SW/PES) completely inactivated all *E. coli*, while it took 15 min for the M3 membrane to completely remove *E. coli*, the same type of bacteria. The differences in the time taken are ascribed to the heavy atom outcome of M2 (In-BP/PES) as compared to M1 (BP/PES), which undergoes fluorescence quickly after excitation to the first excited singlet state. The results for *S. aureus* are much better, with the M3 membrane achieving an almost complete (0.2%) log reduction within 5 min of inactivation under light. After 15 min, all the photosensitizers had completely inactivated all the bacteria. This supports the use of SW to improve the photodynamic antimicrobial activity of neutral photosensitizers as a sustainable and effective approach [25,68,69].

## 4. Conclusions

In conclusion, novel porphyrin-modified PES UF membranes were fabricated via the phase inversion method for an improved bacterial inactivation and filtration performance. The successful synthesis of the porphyrins was confirmed through FTIR, mass spectroscopy, UV-Vis, and fluorescence emission, while the membrane characteristics were studied using FTIR, SEM, CA, porosity, water uptake, and pore size measurements. The modification of the membranes improved the hydrophilicity, fouling resistance, and antibacterial activity under light exposure, specifically those modified by In-BP@SW nanofillers. The In-BP@SW/PES membranes exhibited a high efficacy, as flux recovery ratios of 78.9% and 85% were recorded for the humic acid solution and municipal wastewater, respectively. The In-BP@SW/PES membrane has shown effective antimicrobial photodynamic activity against *S. aureus* and *E. coli* as Gram (+) and Gram (−) strains. At least a 3-log reduction in bacterial survival was attained, showing that they qualify as efficient materials per FDA standards. These membranes offer promising solutions for advanced treatment systems for water that requires bacterial inactivation.

## Figures and Tables

**Table 1:** The casting solutions’ (wt.%) composition.

Membrane ID	PES (wt.%)	NMP (wt.%)	Nanofillers	
			Nanofiller ID	Nanofiller (wt.%)
M0	18	82	-	-
M1	18	81.75	BP	0.25
M2	18	81.75	In-BP	0.25
M3	18	81.75	In-BP@SW	0.25

BP = 5,10,15-tris(5-bromophenyl)porphyrin, In-BP = indium chloride derivative ClIn(III), 5,10,15,tris-(4-bromophenyl)-20-(4-carboxyphenyl)porphyrin, and In-BP@SW = Indium-5,10,15,20-tetracarboxy porphyrin@ single-walled carbon nanotubes.

**Table 2:** The % bacterial viability of M1–M3 for the inactivation effect on *E. coli* and *S. aureus* at 60 min in the light.

% Bacterial Viability						
*E. coli*				*S. aureus*		
	5	10	15	5	10	15
**M1**	69.59	29.83	5.22	7.64	0	0
**M2**	22.58	4.69	0	13.72	0.14	0
**M3**	12.15	0	0	0.20	0	0

**Table 3:** Log reduction values indicating the photoinactivation efficiency of M1–M3 membranes on *S. aureus* and *E. coli*, with 60 min of irradiation using a Thorlabs M425L3 LED.

Log Reduction		
	*S. aureus*	*E. coli*
	Light	Light
**M1**	6.88	5.74
**M2**	7.38	6.76
**M3**	9.79	8.03

## Data Availability

The raw/processed data required to reproduce these findings cannot be shared at this time as the data also form part of an ongoing study.

## Supplementary Materials

The following supporting information can be downloaded at https://www.mdpi.com/article/10.3390/membranes15080239/s1, Figure S1: FTIR spectra for BP and In-BP; Figure S2: Mass spec for PB; Figure S3: Mass spec for In-PB; Table S1: Physicochemical parameters of municipal wastewater before and after treatment.

## Abbreviations

The abbreviations utilized in this manuscript are as follows:

PES	Porphyrin-modified polyethersulfone
UF	Ultrafiltration
CA	Contact angle
MWW	Municipal wastewater
FRRs	Recovery ratios
HA	Humic acid
ROS	Porphyrins generate reactive oxygen species
SWCNTs	Single-walled carbon nanotubes
ACS	Propionic acid
PB	5,10,15-tris(5-bromophenyl)porphyrin
In-PB	5,10,15,tris-(4-bromophenyl)-20-(4-carboxyphenyl)porphyrin Indium-5,10,15,20-tetracarboxy porphyrin (In-PB)
PB@SW	5,10,15,20-tetracarboxy porphyrin@swcnts
In-PB@SW	Indium-5,10,15,20-tetracarboxy porphyrin@swcnts
FTIR	Fourier transform infrared
UV-Vis	Ultraviolet-visible
SEM	Scanning electron microscope
