# Supplementary material for: Porphyrin-Modified Polyethersulfone Ultrafiltration Membranes for Enhanced Bacterial Inactivation and Filtration Performance
Source: Membranes (Basel). 2025 Aug 6;15(8):239. doi: 10.3390/membranes15080239 (PMC12388197; doi:10.3390/membranes15080239)
Supplement: Supplementary file 1 [file membranes-15-00239-s001.zip › membranes-3754576-supplementary.pdf]

Supporting Information

Porphyrin-Modified Polyethersulfone Ultrafiltration Membranes for Enhanced Bacterial Inactivation and Filtration Performance

Funeka Matebese*, Nonkululeko Malomane, Meladi L. Motloutsi, Richard M. Moutloali, and Muthumuni Managa*

Institute for Nanotechnology and Water Sustainability, College of Science, Engineering and Technology, University of South Africa, Florida, 1709, Johannesburg, South Africa.

**Correspondence: matebf@unisa.ac.za; managme@unisa.ac.za*

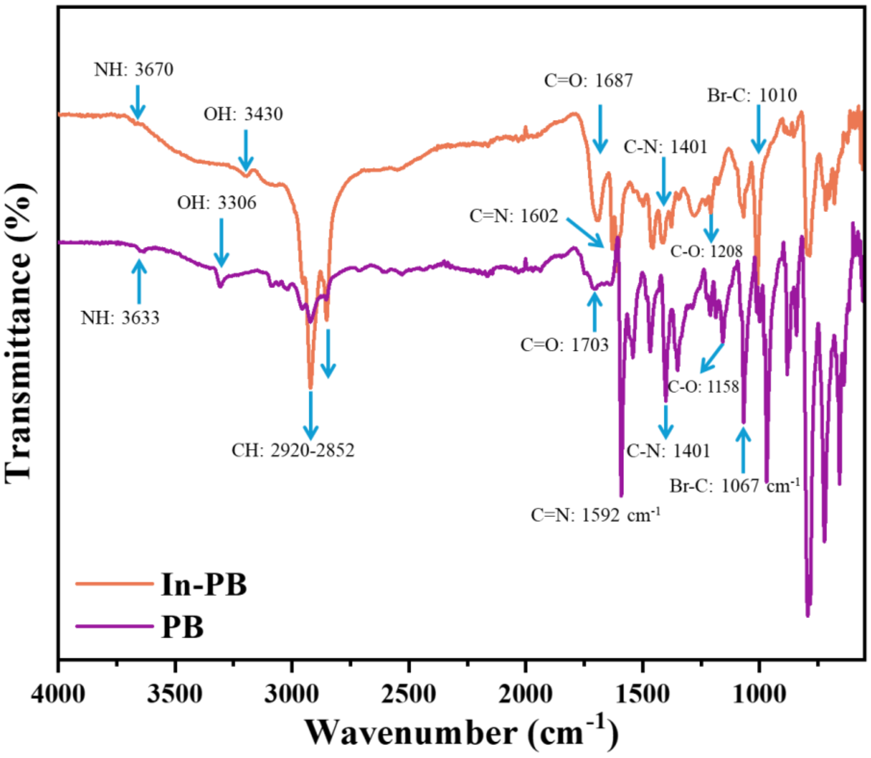

Figure S1. FTIR spectra for BP and In-BP.

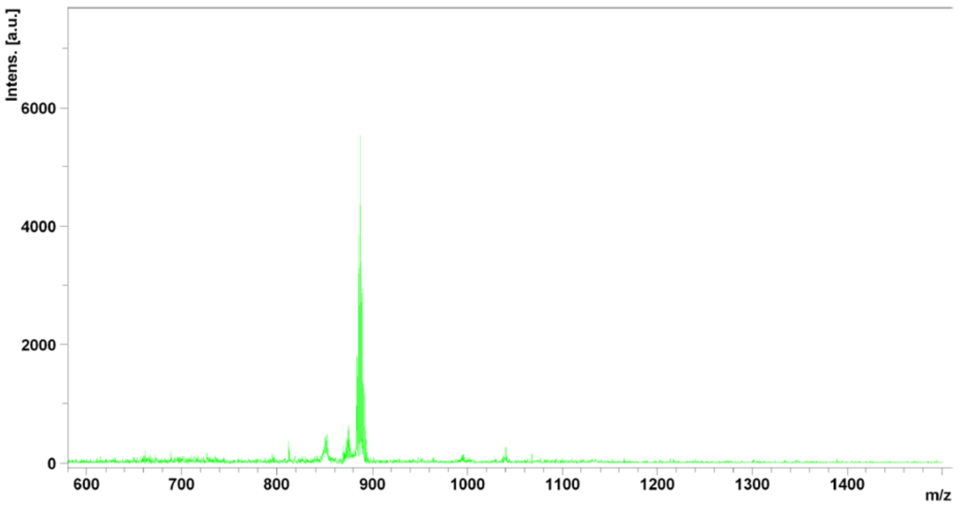

Figure S2. Mass spec for PB.

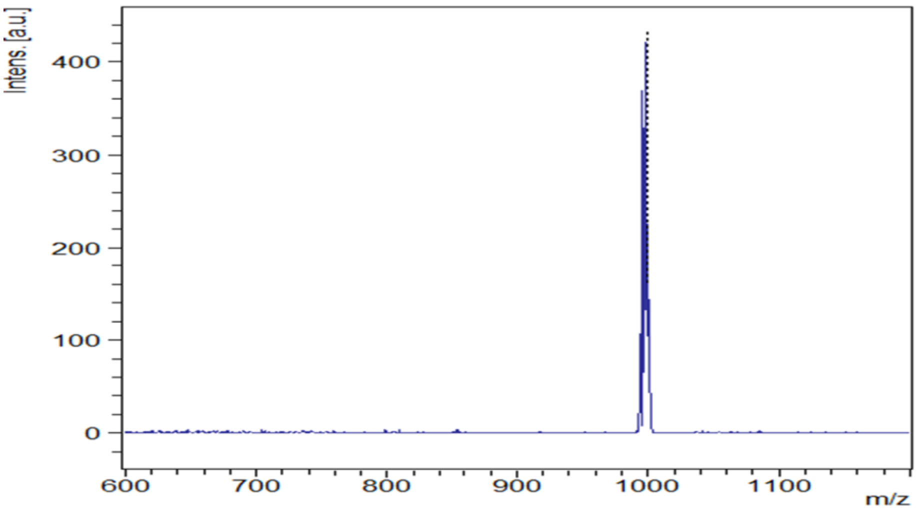

Figure S3. Mass spec for In-PB.

Table S1. Physicochemical parameters of municipal wastewater before and after treatment.

Membrane ID	pH	Turbidity (NTU)	EC (μS.cm^{-1})	TDS (ppm)
Standards	5-9.7	≤5	≤170	≤1200
Raw	7.04	43.64	767	549
M0	7.54	0.87	549	341
M1	7.41	0.14	392	282
M2	7.73	0.04	302	231
M3	7.67	0.00	251	159
